# Exploring Current Molecular Targets in the Treatment of Neovascular Age-Related Macular Degeneration toward the Perspective of Long-Term Agents

**DOI:** 10.3390/ijms25084433

**Published:** 2024-04-17

**Authors:** Serena Fragiotta, Lorena Bassis, Barmak Abdolrahimzadeh, Alessandra Marino, Massimiliano Sepe, Solmaz Abdolrahimzadeh

**Affiliations:** 1 Neurosciences, Mental Health, and Sense Organs (NESMOS) Department, Faculty of Medicine and Psychology, University of Rome Sapienza, 00189 Rome, Italy; s.fragiotta@ausl.latina.it (S.F.); lorena.bassis@uniroma1.it (L.B.); alessandra.marino@uniroma1.it (A.M.); 2 UOC Ophthalmology, Department of Surgical Areas, S.M. Goretti Hospital, 04100 Latina, Italy; m.sepe@ausl.latina.it; 3 North West Ophthalmology, Practiceplus Hospital, Rochdale OL16 2UP, Manchester, UK; barmak.zadeh@practiceplusgroup.com; 4 St. Andrea Hospital, Via di Grottarossa 1035/1039, 00189 Rome, Italy

**Keywords:** age-related macular degeneration (AMD), anti-vascular endothelial growth factor (anti-VEGF), aflibercept, faricimab, brolucizumab

## Abstract

Long-lasting anti-vascular endothelial growth factor (anti-VEGF) agents have become an option to reduce treatment frequency, with ongoing research exploring optimal responses and safety profiles. This review delves into molecular targets, pharmacological aspects, and strategies for achieving effective and enduring disease control in neovascular age-related macular degeneration (AMD). The molecular pathways involved in macular neovascularization, including angiogenesis and arteriogenesis, are explored. VEGF, PlGF, Ang-1, and Ang-2 play crucial roles in regulating angiogenesis, influencing vessel growth, maturation, and stability. The complex interplay of these factors, along with growth factors like TGFβ and bFGF, contributes to the pathogenesis of neovascular membranes. Current anti-VEGF therapies, including bevacizumab, ranibizumab, aflibercept, brolucizumab, and faricimab, are discussed with a focus on their pharmacokinetics and clinical applications. Strategies to achieve sustained disease control in AMD involve smaller molecules, increased drug dosages, and novel formulations. This narrative review provides a comprehensive overview of the molecular targets and pharmacological aspects of neovascular AMD treatment.

## 1. Introduction

Age-related macular degeneration (AMD) represents the leading cause of blindness in people over 65 years of age, with an increasing prevalence in developed countries. As the prevalence of AMD increases, the impact in terms of therapeutic management and prognosis can be considerable, posing significant challenges for healthcare systems [1]. Hallmarks of AMD include drusen and retinal pigment epithelium (RPE) abnormalities, characterizing the early and intermediate stages of the disease [2].

Macular neovascularization (MNV) is a late-stage complication of AMD characterized by the growth of abnormal blood vessels in the macula that can originate either from the choroid or the inner retina [3,4]. Although MNV can remain in a non-exudative state for a variable amount of time, most lesions are associated with exudation, leading to visual decline. Clinically, the exudation is represented by subretinal and/or intraretinal fluid and/or subretinal hyperreflective material (SHRM) that can be observed non-invasively using optical coherence tomography (OCT) [5]. The principal mediator governing neovascularization and vascular permeability is the vascular endothelial growth factor (VEGF), and the inhibition of this factor has revolutionized the treatment of MNV [6].

Before the advent of anti-vascular endothelial growth factor (anti-VEGF) therapies, thermal laser and verteporfin photodynamic therapy were used without any significant chance of improving vision [7]. The introduction of anti-VEGF therapies has led to significant progress in managing neovascular AMD, helping to improve vision and quality of life [8,9]. The use of anti-VEGF medications increased exponentially after Food and Drug Administration (FDA) approval, leading to a dramatic rise in the costs for healthcare systems [10,11]. The need for cost containment has become a serious and controversial public health challenge, with several strategies proposed to minimize the economic impact and maintain the high effectiveness of approved treatments [12]. Anti-VEGF drugs provide anatomical control over exudation, effectively reducing subretinal and intraretinal fluid. However, despite their efficacy in fluid management, neovascular tissue tends to persist after treatment, necessitating repeated injections that can potentially continue indefinitely in many cases [13]. Moreover, some patients may exhibit a suboptimal response to persisting fluid despite intensive treatment, leading to frequent injections [14,15].

Modifying the treatment regimen by introducing a treat and extend (T&E) protocol to increase the cost-effectiveness balance represented one of the first and most important strategies. This proactive regimen consisted of treatment at fixed intervals until clinical remission, proven by the absence of exudative activity on OCT, followed by the progressive extension of treatment intervals. The advantages of this approach included the reduction in treatment burden in terms of visits and injections, reducing recurrences, achieving better long-term vision outcomes, and disease control [16,17,18]. Among the different strategies to overcome the burden of intravitreal anti-VEGF treatment, using long-lasting agents is a promising strategy, attracting the most ongoing research attention. In this regard, increasing the drug durability translates into reducing the need for and frequency of treatments and monitoring visits [19,20]. Obtaining an optimal response in terms of fluid control and visual gain should also be accompanied by an established safety profile [19]. The present narrative review provides an overview of the molecular targets and pharmacological aspects underlying the treatment of neovascular AMD. In this context, exploring the intricate mechanisms and potential strategies for achieving effective and enduring disease control is critical for managing patients with MNV in the current era.

## 2. Molecular Pathways Involved in Macular Neovascularization

**Figure 1:**
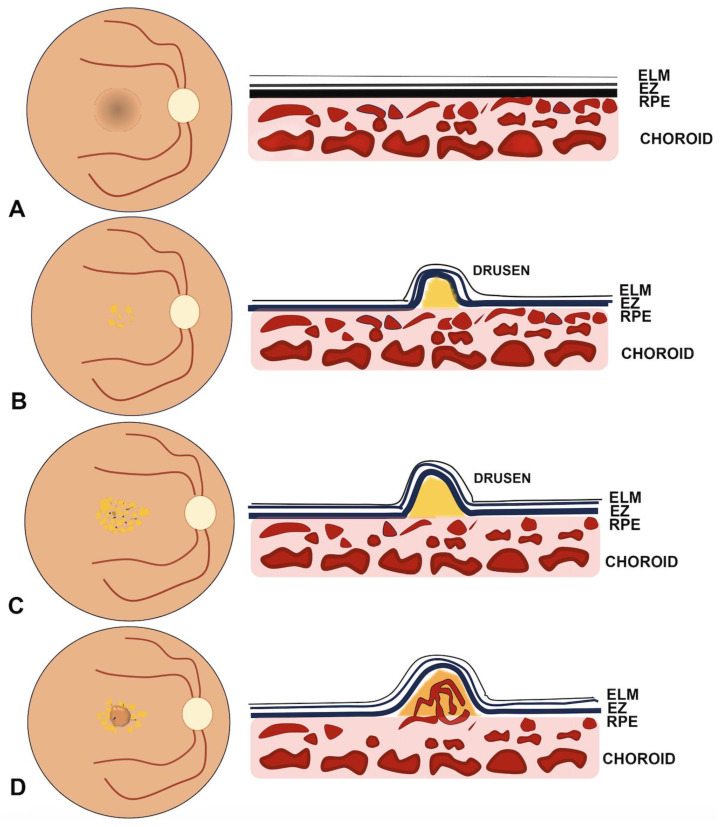
Schematization of clinical staging in age-related macular degeneration (AMD). (**A**) No signs of AMD with normal outer retina and choroid. (**B**) Early AMD is characterized by the presence of drusen ranging between 63 μm and 125 μm without pigmentary changes. (**C**) Intermediate AMD is represented by large drusen > 125 μm and/or pigmentary abnormalities. (**D**) Late AMD with neovascularization arising from choroidal circulation.

The clinical stages of AMD preceding the development of MNV are schematized in Figure 1 [21].

**Figure 2:**
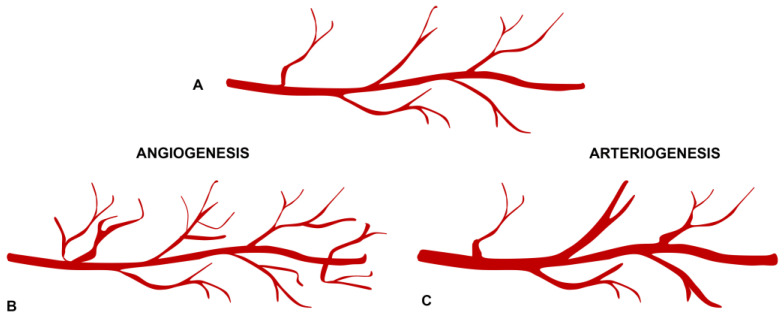
(**A**) Normal vasculature. (**B**) Angiogenesis is characterized by newly formed capillaries sprouting under the vascular endothelial growth factor stimulus. (**C**) Arteriogenesis involves active vascular remodeling without the formation of new vessels; the process is mainly mediated by platelet-derived growth factor (PDGF).

Before delving into the molecular pathways associated with angiogenesis, it is crucial to distinguish between angiogenesis and arteriogenesis in the context of macular neovascularization. Angiogenesis involves the formation of new vessels under VEGF stimulation. This process is characterized by newly formed capillaries forming a neovascular network, usually starting underneath the retinal pigment epithelium (RPE). The newly formed vessels are fragile and, thus, prone to exudation. The angiogenic process can continue until the stimulus represented by VEGF is interrupted (Figure 2) [5].

Arteriogenesis is an alternative angiogenic process wherein pre-existing vessels undergo active remodeling characterized by vasodilatation, leading to the development of collateral vessels, particularly large vessels with reduced capillary perfusion [5,22,23]. The pathophysiology of branching vessel growth involves increasing shear stress, where an increased flow velocity in pre-existing vessels induces structural modifications in the cytoskeleton, adhesion molecules, and growth factors, causing a strong arteriogenic stimulus with collateral vessel development [24,25]. These collateral vessels are less responsive to anti-VEGF therapies [22], as the arteriogenic process is mainly driven by platelet-derived growth factor (PDGF) [26,27].

### 2.1. An Overview of Angiogenic Factors Involved in Angiogenesis

**Figure 3:**
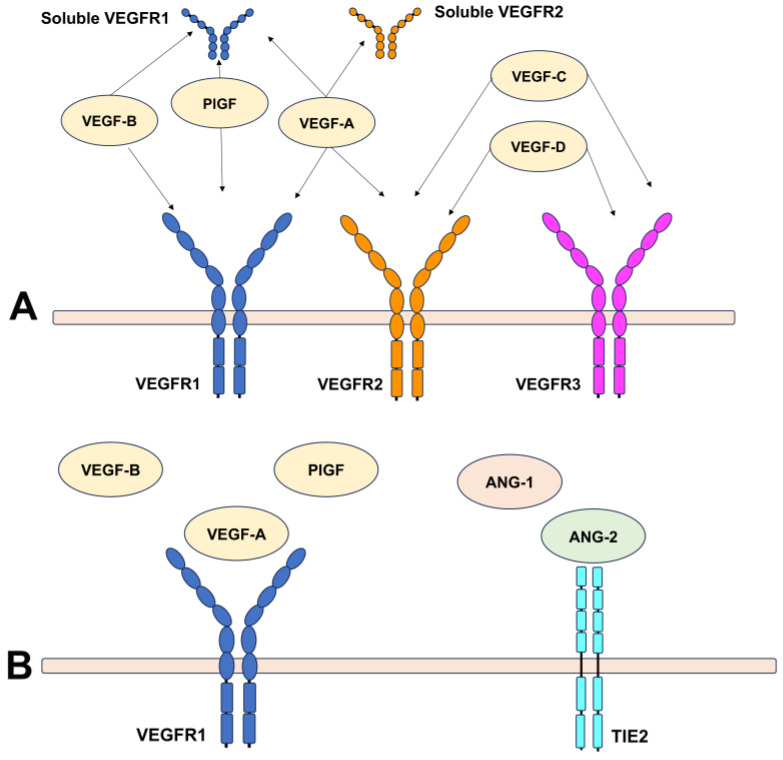
Vascular endothelial growth factor (VEGF) receptors. (**A**) The VEGF family includes several ligands that bind three main receptors, VEGFR1, VEGFR2, and VEGFR3. These receptors are tyrosine kinase receptors, transmembrane proteins constituted of two domains, an extracellular ligand-binding domain and an intracellular kinase domain. VEGFA, placental growth factor (PlGF), and VEGFB bind VEGFR1, while VEGFR2 is activated by VEGFA and both VEGFC and VEGFD, which are also ligands for VEGFR3. (**B**) VEGF mediates its angiogenic effects through the VEGFR1 receptor. Ang-2 competes with Ang-1 for the Tie-2 receptor. The dephosphorylation of VEGFR1 and Tie-2 receptors produces a pro-inflammatory effect decreasing vascular permeability, angiogenesis, and vascular destabilization.

The VEGF family includes several molecules, such as VEGFA, placental growth factor (PlGF), VEGFB, and VEGFC. These VEGF family ligands, to exert their complex biological functions, bind three VEGF receptors in humans, known as VEGFR1 (FLT1), VEGFR2 (KDR/FLK1), and VEGFR3 (FLT4) (Figure 3) [28,29]. VEGF receptors are tyrosine kinase receptors (RTKs), transmembrane proteins constituted of two main domains: an extracellular ligand-binding domain and an intracellular kinase domain [28]. VEGFR1 and VEGRF2 are expressed on the surface of the endothelial cells, while VEGFR3 is presented on lymphatic endothelial cells. VEGFR1 binds VEGFA, PlGF, and VEGFB. This receptor acts as a decoy receptor sequestering VEGFA and thus limiting binding with VEGFR2. VEGFR1 activation via one of its ligands mediates monocyte recruitment, increasing chemotaxis and inflammatory response and also increasing VEGFA and angiogenic mediators, amplifying the angiogenic cascade [30]. The decoy activity of VEGFR1 negatively modulates angiogenesis by sequestering VEGF, thus preventing VEGFR2 from binding to VEGF [30,31]. However, VEGFB can compete with VEGFA for VEGFR1 binding, increasing free VEGFA levels, which can indirectly increase VEGFR2 activation. Likewise, PlGF can bind VEGFR1, competing with VEGFA. This increases VEGFA levels and displaces VEGFA from VEGFR1, but also activates VEGFR2.

VEGFR2 activation is crucial in mediating mitogenic, angiogenic, and permeability effects. This receptor binds VEGFA with a lower affinity than VEGFR1, but also VEGFC and VEGFD [29,30].

VEGFA stimulates vasculogenesis and angiogenesis. Its expression is regulated by oxygen tension, and VEGF mRNA expression increases under hypoxic conditions [32]. VEGF is also involved in the survival and support of the newly formed vessels. In more detail, VEGF stimulates pericyte coating around endothelial cells [33]. Another important function of VEGFA regards the strong vascular permeability, inducing vasodilation and vascular leakage [30,33,34,35]. The interplay between angiogenesis and permeability has been extensively debated. Elevated permeability has the potential to facilitate fibrin extravasation, providing a scaffold for the migration and proliferation of endothelial cells. However, it is crucial to note that vascular permeability can increase independently of angiogenesis. As a result, a clear-cut association between increased permeability and angiogenesis remains unproven [30]. Therefore, anti-VEGF therapies have several implications beyond blocking angiogenesis, such as inducing blood vessel regression and reducing vascular permeability and inflammation.

PlGF plays a pivotal role in orchestrating angiogenesis through different mechanisms, including (a) amplifying the angiogenic activity of VEGFA on endothelial cells, (b) promoting vessel maturation and stabilization by acting on smooth muscle cells, (c) inflammatory cell recruitment contributing to the growth of collateral vessels, and (d) mobilizing vascular and hematopoietic progenitors from the bone marrow, which can contribute to the angiogenic process [36,37]. The actions of PlGF include vessel growth, maturation, and survival. Therefore, these ligands can exert angiogenic effects by acting directly on VEGFR1 and indirectly on VEGFR2 [29]. PlGF influences endothelial growth, migration, and survival via different mechanisms that include (a) direct VEGFR1 binding, (b) VEGFA displacement from transmembrane or soluble VEGFR1, thus increasing the levels of free VEGFA that can bind VEGF, and (c) the formation of heterodimers with VEGFA inducing the development and activation of VEGFR1/VEGFR2 receptor heterodimers [36,37]. PlGF inhibition and VEGFA reduce the reactive microglia and macrophages in the neovascular membrane [29]. Galectin-1 is a galactosidase-binding lectin protein family that binds VEGFR2, contributing to cell adhesion and proliferation, angiogenesis, and immunosuppression [38]. Galectin-1 was found to be overexpressed in laser-induced choroidal neovascularization, corroborating its role in angiogenesis and fibrosis in AMD (Figure 2) [39]. Table 1 summarizes the VEGF family members and their potential involvement in MNV secondary to AMD [29,30,34,35,40].

Angiopoietin 1 (Ang-1) is involved in vascular maturation through the recruitment of pericytes on the newly formed capillaries, while angiopoietin 2 (Ang-2) acts as an antagonist of Ang-1, destabilizing the mature blood vessels [41]. In retinal pathological conditions, the upregulation of Ang-2 competes with Ang-1 for Tie-2 binding. This not only inhibits the vasoprotective effects exerted by Ang-1 but also stimulates pericyte apoptosis, promotes leukocyte adhesion and migration, triggers inflammatory responses, and amplifies angiogenic cytokines, including VEGFA [42]. Notably, although Ang-2 is a natural antagonist of Ang-1 via the angiogenic receptor Tie-2, its function can change according to the VEGF levels. In physiologic vascular remodeling, high levels of Ang-2 in the absence of VEGF are associated with vessel regression. However, the expression of Ang-2 in the context of high VEGF levels contributes to angiogenesis, facilitating the responsiveness to VEGF [43]. Additionally, the inhibition of VEGF appeared to downregulate the levels of Ang-2, leading to normal protein levels in the vitreous and messenger RNA (mRNA) in the retina [44,45]. These findings were interpreted in light of clinical trial results from nesvacumab, a fully human IgG1 monoclonal antibody binding Ang-2 plus aflibercept that exhibited no significant benefits compared to aflibercept alone in treating both neovascular AMD and diabetic macular edema [46,47,48]. The reason was ascribed to the Ang-2 suppression secondary to VEGF inhibition, raising concerns from the FDA regarding the contribution of Ang-2 inhibition in combination with anti-VEGF (Figure 3) [44]. This point seems to be particularly interesting in light of the recently approved faricimab, a drug targeting both VEGF and Ang-2 [49]. However, a direct comparison seems unlikely as the drugs differ in their molecular and pharmacological properties. Future clinical studies are necessary to confirm the preclinical trials’ promising results in a real-life setting, particularly highlighting the potential to achieve long-lasting disease control.

Similarly, the inhibition of VEGF associated with platelet-derived growth factor (PDGF) antagonist failed to meet the primary endpoints compared to anti-VEGF alone, suggesting that VEGF still represents the main pathological driver of angiogenesis [50].

### 2.2. Mediators Isolated in Neovascular Membranes Secondary to AMD

VEGF type A (VEGFA, referred to as VEGF) has been considered a key regulator of angiogenesis in AMD, representing the preferential therapeutical target [6]. The co-occurrence of VEGFA and its receptor has been found in the context of neovascular membranes [51,52,53,54,55]. In surgically removed fibrovascular membranes secondary to AMD, VEGF mRNA was detected in all cases, with higher levels within the neovascular tissue that exhibited a higher degree of inflammation [51]. Despite this, VEGF was the main but not the only mediator of neovascularization, as confirmed experimentally by the evidence that high levels of VEGF cannot induce neovascularization formation alone [56,57]. In human MNV specimens removed during macular translocation surgery, VEGFA, VEGFB, PlGF, and the receptors VEGFR1 and VEGFR2 were detected in all the cases examined through immunohistochemistry [54]. With ischemic retinopathy, both PlGF and VEGF were found to be elevated in the vitreous, and PlGF tended to elevate with the progression of retinopathy [58]. Other angiogenic factors considered in the pathological angiogenesis related to AMD included angiopoietins, which exert their activity by binding a common tyrosine kinase receptor known as Tie-2. Ang-1 and Ang-2 expression has been detected in surgically excised fibrovascular membranes, with Ang-2 overexpression in the highly vascularized areas of the lesion [55,59]. In a study conducted by Hera et al. [55], Ang-1 (16/24 samples) and Ang-2 (10/24 samples) mRNAs were not detectable in any of the examined cases through real-time reverse-transcriptase polymerase chain reaction, while VEGF was present in all the samples.

### 2.3. Angiogenesis and Arteriogenesis in the Pathogenesis of Neovascular Membranes

**Figure 4:**
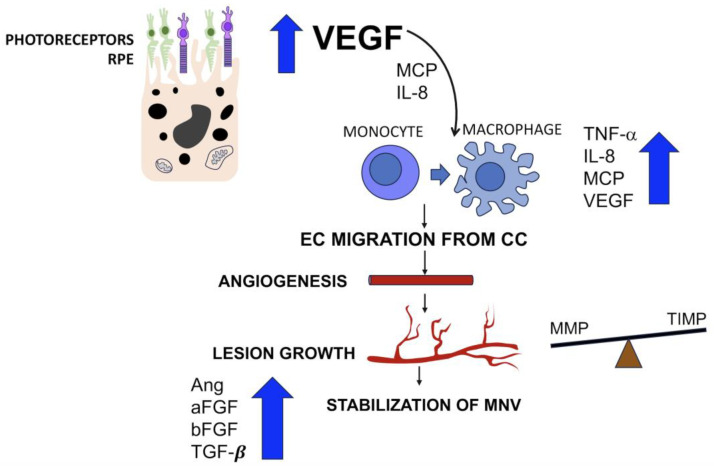
Cascade of events in the angiogenic process. Photoreceptors and retinal pigment epithelium (RPE) produce vascular endothelial growth factor (VEGF), which stimulates monocytes through monocyte colonization protein (MCP) and interleukin-8 (IL-8). Macrophage activation increases the levels of tumor necrosis factor α (TNF-α), increasing the levels of IL-8, MCP, and VEGF. These mediators stimulate endothelial cell (EC) migration from choriocapillaris (CC) and proliferation, leading to new vessel formation (angiogenesis). The newly formed neovascular tissue grows under the balance between matrix metalloproteinases (MMPs), produced by endothelial cells and macrophages, and tissue inhibitors of metalloproteinases (TIMPs), produced by RPE. Vascular remodeling and the subsequent stabilization of the neovascular membrane is favored by the equilibrium of several mediators, including angiopoietins, acidic and basic fibroblast growth factor (aFGF and bFGF), and transforming growth factor beta (TGFβ).

During the initial stages of the angiogenic process, VEGF is produced by photoreceptors and RPE, which concurrently stimulate monocytes through the production of monocyte colonization protein (MCP) and interleukin-8 (IL-8) [51,60,61,62,63]. Macrophages preferentially accumulate in areas of vascular ingrowth, expressing tumor necrosis factor α (TNF-α), thereby amplifying the levels of IL-8, MCP, and VEGF [59,64,65]. The action of TNF-α also enhances the RPE expression of integrins α3 and α5, mediating the RPE migration. At this stage, the endothelial cells proliferate and migrate from choriocapillaris under the VEGF stimulus [66]. The newly formed vessels grow and arborize during the inflammatory active stage [61]. The phase of active growth of the lesions is enhanced by the production of matrix metalloproteinases (MMPs) from the endothelial cells and macrophages, helping the abnormal blood vessels break through tissues. The RPE and other cells produce substances called tissue inhibitors of metalloproteinases (TIMPs) to counteract MMP activity [67]. At this point, the predominant role of VEGF is surpassed by different growth factors that play a role in the expansive phase of choroidal neovascularization. In particular, angiopoietins and their receptors (Tie-1, Tie-2) are variably expressed [55,59,68], and acidic and basic fibroblast growth factor (aFGF and bFGF) produced by the RPE and vascular endothelium contribute to the vascular remodeling of the neovascular lesion, as well as transforming growth factor beta (TGFβ) [69,70]. The interaction between the TGFβ and bFGF released by macrophages is essential for neovascular membrane formation. TGFβ increases the inflammatory response through chemotaxis and induces dedifferentiation of RPE into fibroblasts, while bFGF binds the extracellular matrix and induces angiogenesis [70]. The subsequent stage is characterized by a stabilization of the neovascular membrane, reaching an equilibrium among all the factors involved, including matrix metalloproteinases (MMPs), angiopoietins, VEGF, pigment epithelium-derived factor, platelet-derived growth factor, plasminogen, fibrin, and various other factors [59,71,72,73]. For further details, see Figure 4. The last stage is characterized by a shift towards antiangiogenic, anti-proteolytic, and anti-migratory activity, leading to an involutional stage of the neovascular membrane, which can be recognized clinically with a disciform scar. In this stage, the role of RPE appears predominant with the increased production of TGFβ and TIMP3 [66].

## 3. Current Anti-VEGF Therapies for MNV

Several anti-VEGF drugs have been used for the treatment of neovascular AMD. Bevacizumab was the first anti-VEGF drug introduced in 2004 for the treatment of metastatic colon carcinoma. However, this drug never received FDA approval for ophthalmic use, even if it was widely used “off-label”, repackaging the available intravenous formulation [10,12]. The first anti-VEGF drug approved for ophthalmic use to treat neovascular AMD was pegaptanib sodium (Macugen, Pfizer, New York, NY, USA) approved in 2004, followed by ranibizumab in 2006 (Lucentis, Genentech, South San Francisco, CA, USA), and aflibercept in 2011 (Eylea, Regeneron, New York, NY, USA). Recently released drugs include brolucizumab (Beovu, Novartis Pharmaceuticals, Ltd., London, UK) in 2019, and the latest drug receiving FDA approval for treating neovascular AMD, faricimab (Vabysmo^TM^, Roche, Genentech, South San Francisco, CA, USA), was approved in 2022 [10,74,75]. A comparison by the Age-related Macular Degeneration Treatments Trials (CATT) Research Group compared the effects of ranibizumab and bevacizumab administered on a monthly basis or as needed, demonstrating similar effects on visual acuity but a higher rate of serious adverse events for patients treated with bevacizumab [13,14]. The utilization of bevacizumab off-label has opened a significant and multifaceted debate, traversing regulatory, scientific, and social levels. The discussion surrounding its off-label use remains a topic of ongoing controversy that involves considerations of safety, efficacy, regulatory compliance, ethical implications, and the potential impact on healthcare practices [76]. Noteworthy is that the clinical trials comparing different anti-VEGF therapies are non-inferiority studies, limiting a direct comparison among the existing drugs [77,78,79,80,81]. Table 2 summarizes the commercialized therapeutic agents for the treatment of MNV.

### 3.1. Bevacizumab

Bevacizumab is a full-length humanized anti-VEGF monoclonal antibody with a binding affinity to VEGF comparable to the original antibody (Kd~0.5 nM). Bevacizumab (molecular weight 149 KDa) binds VEGFA isoforms and bioactive proteolytic fragments [32]. As mentioned above, bevacizumab was approved for oncological use, but it is widely used worldwide for the treatment of wet AMD at a dosage of 1.25 mg/50 muL [10,76]. Vitreous half-life was variable between 2.5 and 7.3 days with a mean of 4.9 days in a series of three patients who underwent pars plana vitrectomy after injection [82]. In another study, intravitreal bevacizumab exhibited a two-compartment model with a first distribution phase followed by an elimination phase, with an estimated half-life of 6.7 days [83]. The minimum effective drug concentration to block VEGFA was 500 ng/mL, estimating that an appropriate interval for repeated injection to maintain this therapeutic level was 6–7 weeks [83]. The serum half-life of bevacizumab after three intravitreal injections was 18.7 days, exhibiting a serum concentration of 1.58 nM that was higher than the inhibitory concentration (IC50) of 0.668 nM, suggesting a possible relationship with the higher rate of systemic complications after intravitreal bevacizumab [84].

### 3.2. Ranibizumab

Ranibizumab is a fragment of monoclonal antibody without an Fc region (molecular weight 48 KDa) that binds all subtypes of VEGFA. The therapeutic dose is 0.5 mg administered intravitreally [8,85]. Ranibizumab was developed as an affinity-matured antigen-binding fragment (Fab) to improve the diffusion from the vitreous into the tissue targets (retina and choroid) [31]. The vitreous half-life of ranibizumab was 9 days and 7.19 days in the aqueous humor [86,87]. Besides ocular clearance, ranibizumab has 5- to 20-fold higher biological activity than bevacizumab [31,87]. The vitreous slowly releases ranibizumab into the systemic circulation, where it is readily eliminated with an intrinsic half-life of 2 h and a concentration 90.000 times lower than the vitreous compartment [86].

The port delivery system (PDS) with ranibizumab is a new intraocular delivery system developed for a long-acting continuous release of the drug into the vitreous. The PDS is constituted of an implant body with a reservoir containing the drug (ranibizumab 100 mg/mL) with a release control element that progressively releases the drug into the vitreous, an extrascleral flange that anchors the implant to the sclera, and a self-sealing septum where the drug can be refilled [88]. Controlled release of ranibizumab is achieved via passive diffusion, which depends on the drug concentration within the implant [89]. The estimated half-life of the implant was 99 days in vitro, but a recent clinical trial on humans with AMD corroborated a similar half-life of 106 days [90]. The FDA-approved PDS implant represents a long-acting drug delivery system engineered to release the drug over 6 months continuously [89]. The PDS was approved in 2021 by the FDA in neovascular AMD with a proven response to at least two intravitreal injections of an anti-VEGF drug, with a dosage of 100 mg/mL and fixed 24-week refill exchanges [90].

### 3.3. Aflibercept

Aflibercept is a soluble decoy receptor developed using Trap technology, which involves the fusion of antibody components derived from multiple endogenous receptors. Aflibercept (molecular weight 115 KDa) consists of all human DNA sequences of the second domain of the VEGFR1 immunoglobulin (Ig) and the third domain of human VEGFR2 Ig that is fused to the Fc region of IgG1 [91]. Aflibercept is then able to bind not only VEGF with a great affinity but also other ligands, owing to the different domains that include VEGFB, PlGF, and galectin-1 [38,92,93]. The multitarget activity of aflibercept is able to reduce neovascular activity, vascular permeability, and fluid accumulation through the inhibition of VEGFA and -B [29,93]. Additionally, aflibercept influences the inflammatory response by suppressing monocyte recruitment and activation by inhibiting PlGF and galectin-1 [29,93]. A downregulation of Ang-2 has also been reported after aflibercept administration, probably as an indirect consequence of solid anti-VEGF inhibition [44]. Factors implicated in the favorable clinical outcomes with aflibercept include its higher binding affinity, interaction with multiple molecular targets, and distinctive ocular pharmacokinetics [29].

Vitreous half-life was 9.1 days and 11 days in the aqueous humor [92,94]. Moreover, aflibercept binds VEGF with a higher affinity with a dissociation constant of 0.49 pM, compared to either bevacizumab (Kd = 58 pM) or ranibizumab (Kd = 46 pM). Once bound to VEGF, the complex is stable with almost no release of VEGF; ranibizumab and bevacizumab require a 10- to 100-fold greater molar concentration to achieve equivalent inhibition of VEGF [93]. In a theoretical model, the biological activity of aflibercept 10 weeks from the injection is comparable to ranibizumab at 30 days. The prolonged biological activity was explained by the higher binding activity and presumed longer intravitreal half-life [95]. The serum half-life was 11.4 days after three monthly injections of 2 mg of aflibercept [96,97].

### 3.4. Brolucizumab

Brolucizumab is a humanized single-chain antibody fragment (scFv) that binds all the isoforms of VEGFA. The molecule is an antibody molecule’s smallest (28 kDa) functional subunit. The small structure has been considered an advantage in tissue penetration and bioavailability, leading to prolonged clinical activity and optimal fluid control [98,99]. This was also hypothesized to be an advantage regarding immunogenicity, but a difference in the inflammation profile of brolucizumab has been noted compared to other anti-VEGF compounds, particularly in cases with occlusive vasculitis in addition to mild-to-moderate intraocular inflammation. The reasons for the increased immunogenicity of the drug are still not completely understood, but type 3 and/or type 4 immunogenic responses with the formation of anti-drug antibodies have been hypothesized [100]. The clinical dose for treating neovascular AMD is 6 mg with a concentration of up to 120 mg/mL in a single 50 μL intravitreal injection. At this concentration, it has been estimated that the anti-VEGF binding capacity is 11 and 22 times higher than that of aflibercept and ranibizumab [101]. Free brolucizumab concentration increased in the vitreous 21 days after the third injection. In order to mitigate the potential immunogenicity resulting from drug accumulation, the use of a monthly regimen has been discouraged, leading to the termination of clinical trials [100]. In a rabbit model, the vitreous half-life of brolucizumab was 3.1 days, similar to ranibizumab, which showed 3.15 days of half-life but was inferior to aflibercept, which presented a vitreous half-life of 5.63 days. The same model reveals a two- to three-fold higher volume distribution of brolucizumab compared to aflibercept and ranibizumab [102]. The serum half-life was 51 h with a dose 3500-fold lower than that in the vitreous [101].

### 3.5. Faricimab

Faricimab is a bispecific molecule bound to an optimized Fc fragment with a molecular weight of 150 kDa [75,80,81]. Faricimab, designed as a human IgG1-like CrossMab, incorporates an engineered Fc region to reduce immunogenicity and systemic half-life. The modified Fc region, achieved through aminoacidic substitutions, has eliminated its binding capacity to human Fc receptors, thereby abolishing the Fc-mediated effector functions, such as antibody-dependent cytotoxicity, antibody-dependent cell phagocytosis, and complement-dependent cytotoxicity [42,103].

The molecule was designed with one antigen-binding site for VEGF and another for Ang-2 with an affinity comparable to ranibizumab (3.3 vs. 3.1 nM) [103]. The bispecific activity of the drug is able to simultaneously inhibit VEGF, acting on neovascularization and vascular permeability, and Ang-2, which promotes vascular leakage and abnormal vessel structure [41,71,104]. The rationale of dual inhibition comes from preclinical evidence demonstrating a sustained reduction in vascular leakage and inflammatory pathways when compared to the sole inhibition of VEGFA [79].

The therapeutic dose of faricimab is 6 mg, administered intravitreally [80,81]. Vitreous half-life was estimated at 7.5 days; the drug reached a plasmatic peak after 2 days without systemic accumulation after repeated injections [49].

## 4. Strategies to Achieve Sustained Disease Control in AMD

New neovascular AMD agents were developed to achieve longer and sustained disease control. With an extended regimen, treatment intervals can be extended up to 16 weeks in most cases using the available products, such as aflibercept, brolucizumab, and faricimab [81,105,106,107,108]. Nevertheless, current efforts are concentrated on addressing the unmet needs stemming from anti-VEGF use. The ultimate objective is to attain prolonged disease control with reduced injection frequency and an optimal safety profile. Key challenges in this context include suboptimal responses to treatment, the associated burden of anti-VEGF treatments, and the safety profile [19]. Prolonging the drug action in the vitreous is one of the main strategies to improve disease control and reduce treatment frequency. The first strategy consists of using smaller molecules, such as brolucizumab (26 kDa), increasing the clinical dose in the same amount of volume that can be injected into the eye [101,109]. An increased binding affinity demonstrated a greater potency with a more significant VEGF inhibition into the eye [93]. Another strategy is to increase the drug dosage of an existing anti-VEGF drug, which was recently developed with a new formulation of aflibercept 8 mg in a volume of 0.07 mL. This strategy was deemed promising in reducing treatment frequency and prolonging the dosing regimen [110]. Quadrupling a drug dose is expected to add two extra half-lives, extending the drug duration [109]. Another approach consisted of using multitarget molecules such as faricimab that are able to bind different ligands like VEGF and Ang-2. Clinical trials have demonstrated a non-inferiority of T&E intravitreal faricimab compared to aflibercept in a fixed dose interval of 8 weeks, with 80% of patients receiving injections every 12 or 16 weeks [49,75,81,110].

Other strategies for long-standing disease control include the pegylation of drugs to increase molecular size, limit diffusion, extend half-life, and minimize the frequency of injections, but the modification of charge and the co-administration of human serum albumin with a FaBa fragment could also increase the intraocular life [109]. Among these strategies, alternative administration routes are also under exploration. For instance, sustained drug delivery with a PDS that released ranibizumab directly into the vitreous cavity was able to provide sustained disease control for up to 6 months [88,89,90]. Some concerns exist regarding the potential side effects in the long term due to bleb formation or conjunctival erosions [109].

## 5. Conclusions

In conclusion, AMD poses a significant public health challenge as a leading cause of blindness in the elderly. The advent of anti-VEGF therapies has transformed the management of neovascular AMD, offering substantial progress in vision improvement and quality of life. However, the substantial economic impact and the need for cost containment have sparked controversy and prompted innovative strategies. Long-lasting agents are notable approaches aimed at achieving a balance between cost-effectiveness and therapeutic efficacy. Understanding the intricate molecular interplay between these mediators holds significant importance in maintaining the delicate equilibrium between neoangiogenesis and vascular regression. Careful consideration of these dynamics is essential for a comprehensive knowledge of pathologic neovascularization to then develop an appropriate therapeutic strategy.

Angiogenesis represents an intricate process involving several factors, culminating in the formation of new blood vessels or neovessels. These newly formed capillaries are prone to exudation and bleeding, leading to irreversible and profound functional morphology. The distinction between angiogenesis and arteriogenesis should not be overlooked, considering that these processes present a distinct and peculiar pathogenesis with different growth factors involved. The VEGF family still appears to be the key regulator of angiogenesis, being the most effective target for disease control. Most of the efforts are made to prolong the drug duration in the eye and potentiate the effect of anti-VEGF by blocking other mediators. In this regard, both aflibercept and faricimab have demonstrated significant efficacy, reaching 16-week interval extensions with a good safety profile. Although the dual inhibition of VEGF and Ang-2 has been debated, given the experimental and past clinical trial evidence, the preliminary results from trials are encouraging, making real-life experience with faricimab critical. Still, a step forward to a greater interval extension with sustained control of the disease between intervals and optimal safety is highly desirable. Promising strategies aim to prolong the drug action in the eye, using smaller molecules, increased drug dosage or concentration, or multifunctional agents or modifying existing anti-VEGF agents to reduce their clearance. These strategies reflect ongoing efforts to enhance treatment outcomes and reduce the burden on patients and healthcare systems. As research continues to unravel the complexities of AMD pathogenesis, the pursuit of sustained disease control with optimal safety profiles remains a central focus. 

## Figures and Tables

**Table 1:** Vascular endothelial growth factor (VEGF) family members involved in macular neovascularization.

Ligands	Receptor	Biological Function	Role in AMD
VEGFA	VEGFR1, VEGFR2	Angiogenesis Migration and proliferation of EC Vascular permeability Inflammation	New vessel formation in NV Fluid extravasation Support of neovascular membrane Inflammatory response
PlGF	VEGFR1	Increase VEGFA levels Proliferation, migration, and survival of EC Proliferation and recruitment of vascular smooth cells	Stimulate vessel growth and remodeling of NV Vessel maturation
VEGFB	VEGFR1	Influence VEGFA levels Inflammation Vascular remodeling	Stimulate vessel growth and remodeling of NV
VEGFC	VEGFR2, VEGFR3	Regulates lymphatic vessels	None
VEGFD	VEGFR2, VEGFR3	Regulates lymphatic vessels	None

VEGF: vascular endothelial growth factor; PlGF: placental growth factor.

**Table 2:** Molecular characteristics of the anti-vascular endothelial growth factor (anti-VEGF) drugs and main characteristics of the different anti-VEGF intravitreal injections.

Characteristics	Bevacizumab	Ranizibumab	Aflibercept	Brolucizumab	Faricimab
Class	Monoclonal Ab	Fab fragment	Fusion protein	scFv	Monoclonal Ab bispecific
Target	VEGFA	VEGFA	VEGFA, PlGF, VEGFB, galectin-1	VEGFA	VEGFA, Ang-2
Dose	1.25 mg	0.5 mg	2 mg	6 mg	6 mg
Molecular weight	149 kDa	48 kDa	115 kDa	26 kDa	150 kDa
Dissociation constant	58 pM	46 pM	0.49 pM	28.4 pM	3 nM
Vitreous half-life (days)	4.9	9	9.1	3.1	7.5

Ab: antibody; Fab: fragment antigen binding; scFv: single-chain variable fragment; kDa: kilodaltons; pM: picomolar; nM: nanomolar.

## Data Availability

Not applicable.
